# Age-Dependent Susceptibility to Alcohol-Induced Cerebral Artery Constriction

**DOI:** 10.4303/jdar/236002

**Published:** 2016-11-27

**Authors:** Anna N. Bukiya, Olga Seleverstov, Shivantika Bisen, Alex M. Dopico

**Affiliations:** Department of Pharmacology, College of Medicine, The University of Tennessee Health Science Center, Memphis, TN 38103, USA

**Keywords:** brain vessel, MaxiK channel, vascular smooth muscle, cholesterol, filipin

## Abstract

**Background:**

Age has been recognized as an important contributor into susceptibility to alcohol-driven pathology.

**Purpose:**

We aimed at determining whether alcohol-induced constriction of cerebral arteries was age-dependent.

**Study design:**

We used rat middle cerebral artery (MCA) in vitro diameter monitoring, patch-clamping and fluorescence labeling of myocytes to study an age-dependent increase in the susceptibility to alcohol in 3 (50 g), 8 (250 g), and 15 (440 g) weeks-old rats.

**Results:**

An age-dependent increase in alcohol-induced constriction of MCA could be observed in absence of endothelium, which is paralleled by an age-dependent increase in both protein level of the calcium-/voltage-gated potassium channel of large conductance (BK) accessory *β*1 subunit and basal BK channel activity. Ethanol-induced BK channel inhibition is increased with age.

**Conclusions:**

We demonstrate an increased susceptibility of MCA to ethanol-induced constriction in a period equivalent to adolescence and early adulthood when compared to pre-adolescence. Our work suggests that BK *β*1 constitutes a significant contributor to age-dependent changes in the susceptibility of cerebral arteries to ethanol.

## 1. Introduction

Alcohol drinking is widespread affecting virtually any age group in the US. For instance, 8.7 million of American youth aged 12–20 y reported that they drank alcohol in the past month (National Survey on Drug Use and Health (NSDUH) 2014. Available at http://www.samhsa.gov/data/sites/default/files/NSDUH-DetTabs2014/NSDUH-DetTabs2014.htm#tab2-15a). When a wider age group that includes older individuals is considered, around 92% of US adults reported excessive alcohol consumption in the past 30 days (http://www.cdc.gov/alcohol/fact-sheets/binge-drinking.htm). Thus, excessive alcohol consumption represents a major public health problem. In addition to societal, psychological, and behavioral disruption, alcohol intake favors and/or triggers specific pathological conditions, including cerebrovascular disease [1, 2]. Age has been increasingly recognized as an important determinant of susceptibility to alcohol intake and the resulting behavioral changes [3, 4, 5, 6, 7]. Moreover, the role of age as a factor in alcohol-related organ pathology has also been increasingly recognized [8, 9, 10, 11]. However, whether alcohol-induced effects on cerebrovascular function are age-dependent remains unknown.

Studies in animal models and humans show that alcohol (ethanol) deprives the brain of adequate blood flow [12]. This ethanol effect has been attributed to a concentration-dependent (10–100 mM) ethanol-induced constriction of cerebral arteries [12, 13, 14, 15]). Middle cerebral arteries control local vascular resistance and represent a valid model for studying ethanol effect on cerebral artery diameter [15, 16, 17]. In a rat model widely used to mimic human cerebral artery function, it has been demonstrated that ethanol-induced cerebrovascular constriction is caused by drug-induced inhibition of Ca^2+^- and voltage-gated K^+^ channels of large conductance (BK channels) in vascular smooth muscle [13]. These channels are critical regulators of arterial diameter and myogenic tone: BK channel activation in vascular smooth muscle results in outward potassium currents, which negatively feedback on depolarization-induced Ca^2+^-entry and thus reduce smooth muscle contraction [18, 19, 20]. Therefore, BK channel inhibition by ethanol results in cerebral artery constriction [13, 14]. BK channels in cerebral artery myocytes consist of channel-forming *α* or slo1 and accessory *β*1 subunits [21, 22, 23]. The latter is critical for ethanol-induced constriction, as ethanol effect is absent in cerebral arteries from *KCNMB1* knock-out (*β*1 subunit-lacking) mouse cerebral arteries [14].

In the present study, we document for the first time an age-dependent increase in the degree of ethanol-induced constriction of rat cerebral arteries. This increase could be observed in absence of a functional endothelium. Our study also demonstrates that the progressive increase in the susceptibility of cerebral arteries to ethanol is paralleled by an age-dependent increase in BK *β*1 proteins levels while unrelated to changes in BK *α* protein levels or cerebral artery myocyte cholesterol level, the latter being a key determining of the response of cerebral artery BK channels to ethanol and eventual vasoconstriction [15]. Within the physiological range of voltage and Ca^2+^, BK *β*1 subunits lead to increased BK current, and also mediate ethanol-induced channel inhibition and vasoconstriction [14, 22, 23]. Consistently, our results show that aged-induced increase in BK *β*1 protein levels are paralleled by an increase in basal BK channel activity and degree of ethanol-induced BK channel inhibition in membrane patches from freshly isolated cerebral artery myocytes. Our work suggests that BK channel *β*1 protein may represent a significant player that contributes to age-dependent changes in the susceptibility of cerebral arteries to ethanol-induced constriction.

## 2. Materials and methods

### 2.1. Ethical aspects of research

The care of animals and experimental protocols were reviewed and approved by the Animal Care and Use Committee of the University of Tennessee Health Science Center, which is an institution accredited by the Association for Assessment and Accreditation of Laboratory Animal Care.

### 2.2. Cerebral artery diameter measurement

Male Sprague-Dawley rats of three age groups (3, 8, and 15 weeks weighting *≈* 50 g, *≈* 260 g, and *≈* 440 g, respectively) were decapitated using a guillotine. Rat middle cerebral arteries were isolated on ice under microscope (Nikon SMZ645; Nikon, Tokyo, Japan) and cut into 1–2 mm-long segments. When required by the experimental protocol, their endothelium was removed by passing an air bubble into the vessel lumen for 90 s before vessel cannulation [15]. Intact and de-endothelialized arterial segments were cannulated at each end in a custom-made perfusion chamber. Using a Dynamax RP-1 peristaltic pump (Rainin Instruments, CA, USA), the chamber was continuously perfused at a rate of 3.75 mL/min with physiologic saline solution (PSS) (mM): 119 NaCl, 4.7 KCl, 1.2 KH_2_PO_4_, 1.6 CaCl_2_, 1.2 MgSO_4_, 0.023 EDTA, 11 glucose, and 24 NaHCO_3_. The PSS was equilibrated at pH 7.4 with a 21/5/74% mixture of O_2_/CO_2_/N_2_ gases and maintained at 35–37 °C. Arteries were evaluated with a CCD camera (Sanyo VCB-3512T; Sanyo Electric Co., Osaka, Japan) attached to an inverted microscope (Nikon Eclipse TS100; Nikon). The artery external wall diameter was measured using the automatic edge-detection function of IonWizard software (IonOptix, MA, USA) and was digitized at 1 Hz.

Steady-state changes in intravascular pressure were achieved by elevating an attached reservoir filled with PSS and were monitored using a pressure transducer (Living Systems Instrumentation, VT, USA). Arteries were first pressurized at an intravascular pressure of 10 mm Hg for 15 min. Then, intravascular pressure was increased to 60 mm Hg and held steady throughout the experiment. Experimental procedures were started after arteries developed myogenic tone in response to 60 mm Hg. Drugs were dissolved to make stock solutions, diluted in PSS to their final concentration, and applied to the artery with PSS perfusion. The effect of a drug application was evaluated at the time it reached a maximal, steady level. During experimentation on de-endothelialized arteries, the absence of endothelium was confirmed by the absence of the vessel’s response to an endothelium-dependent vasodilator (10 *μ*M acetylcholine) after artery pressurization [13, 24].

### 2.3. Myocyte isolation and patch-clamp electrophysiology recordings

Myocytes were isolated from middle cerebral arteries of male Sprague-Dawley rats as previously described by our group [25]. In brief, arteries were placed into ice-cold dissociation medium (DM) with the following composition (mM): 0.16 CaCl_2_, 0.49 EDTA, 10 HEPES, 5 KCl, 0.5 KH_2_PO_4_, 2 MgCl_2_, 110 NaCl, 0.5 NaH_2_PO_4_, 10 NaHCO_3_, 0.02 phenol red, 10 taurine, and 10 glucose. Individual myocytes were enzymatically isolated by a 2-step enzymatic digestion with 0.03% papain followed by 2% collagenase (26.6 units/mL). Artery tissue-containing DM was pipetted using a series of borosilicate Pasteur pipettes having firepolished, diminishing internal diameter tips. The procedure rendered a cell suspension containing relaxed, individual myocytes that was stored in ice-cold DM containing 0.06% soybean trypsin inhibitor and 0.06% BSA. Myocytes were used for electrophysiology up to 4 h after isolation.

Immediately before recording, myocytes were dispersed and left to settle for 10–15 min on a poly-lysine-treated plastic dish. For patch-clamp experiments, both bath and electrode solutions contained (mM): 130 KCl, 5 EGTA, 2.28 MgCl_2_, 15 HEPES, and 1.6 N-(2-Hydroxyethyl) ethylenediaminetriacetic acid (HEDTA), pH 7.35. The nominal free Ca^2+^ was calculated with MaxChelator Sliders (C. Patton, Stanford University), and free Ca^2+^ in solution was adjusted to 30 *μ*M by adding CaCl_2_. Nominal free Ca^2+^ values have been validated experimentally using Ca^2+^-selective electrodes [26]. An agar bridge with chloride was used as a ground. After excision from the cell, the membrane patch was exposed to a stream of either ethanol-free or ethanol-containing bath solution. Solutions were applied onto the patch cytosolic side using a pressurized, automated OctaFlow system (ALA Scientific Instruments, NY, USA) via a micropipette tip with an internal diameter of 100 *μ*m. Experiments were performed at room temperature (20–22 °C).

Ionic current was recorded using an EPC8 amplifier (HEKA, Lambrecht, Germany) at 1 kHz. Data were digitized at 5 kHz using a Digidata 1320A A/D converter and pCLAMP 8.0 (Molecular Devices, Sunnyvale, CA, USA). The product of number of channels in the patch (N) and channel open probability (Po) was used as an index of channel steady-state activity. NPo was obtained using a built-in option in Clampfit 9.2 (Molecular Devices) from ≥ 20 s of gap-free recording under each condition.

### 2.4. Immunofluorescence imaging

Procedures on myocytes from each of the animal groups (50 g, 250 g, and 440 g) were performed simultaneously. Freshly dispersed myocytes were fixed in 4% paraformaldehyde and treated with permeabilizing/blocking solution containing 20% goat serum, 2% bovine serum albumin, and 0.1% Triton X-100 for 30 min. Slips were incubated with mouse monoclonal anti-BK *α* (channel-forming) subunit antibody (Neuromab, CA, USA) and rabbit polyclonal anti-BK *β*1 subunit antibody (Thermo Scientific, MA, USA) at 4 °C overnight. Negative control staining was performed with each antibody as previously described [27]. The day following overnight incubation with anti-BK subunit antibodies, slips were washed and incubated with preabsorbed Alexa-488-conjugated antimouse and Cy5-conjugated antirabbit secondary antibodies at room temperature in the dark for 2 h. Upon washout, myocytes were stained with filipin-containing phosphate buffer solution (25 microg/mL prepared from stock solution of 10 mg/mL filipin in dimethyl sulfoxide) for 2 h at room temperature in the dark.

Slips were mounted using ProLong AntiFade kit (Invitrogen, CA, USA) and sealed using clear nail polish. Immunofluorescence images were obtained sequentially using 405, 488, and 635 laser lines of an FV-1000 laser scanning confocal system (Olympus, PA, USA). Background fluorescence was subtracted from final fluorescence intensity at each channel of data acquisition. Membrane areas were defined by an overlay of the fluorescence image with the image of the myocyte in visible light.

### 2.5. Chemicals

Ultrapure ethanol (ethyl alcohol) was purchased from American Bioanalytical (Natick, MA, USA). All other chemicals were purchased from Sigma-Aldrich (St. Louis, MO, USA). For paxilline-containing solution, 114.6 mM paxilline stock was prepared in dimethyl sulphoxide (DMSO). Stock was further diluted with PSS to a final concentration of 1 *μ*M paxilline.

### 2.6. Data Analysis

Final plotting, fitting, and statistical analysis of data were conducted using Origin 8.5.1 (OriginLab) and InStat 3.0 (GraphPad). Statistical analysis was conducted using either one-way ANOVA analysis of variance and Tukey’s multiple comparison test or Student’s *t*-test according to the experimental design. *P < .*05 was considered statistically significant. Data are expressed as the mean *±*SEM, and *N* = number of arteries. Each artery diameter or cholesterol/protein measurement was obtained from a separate animal.

## 3. Results

### 3.1. Ethanol-induced cerebral artery constriction is increased with age

**Figure 1:**
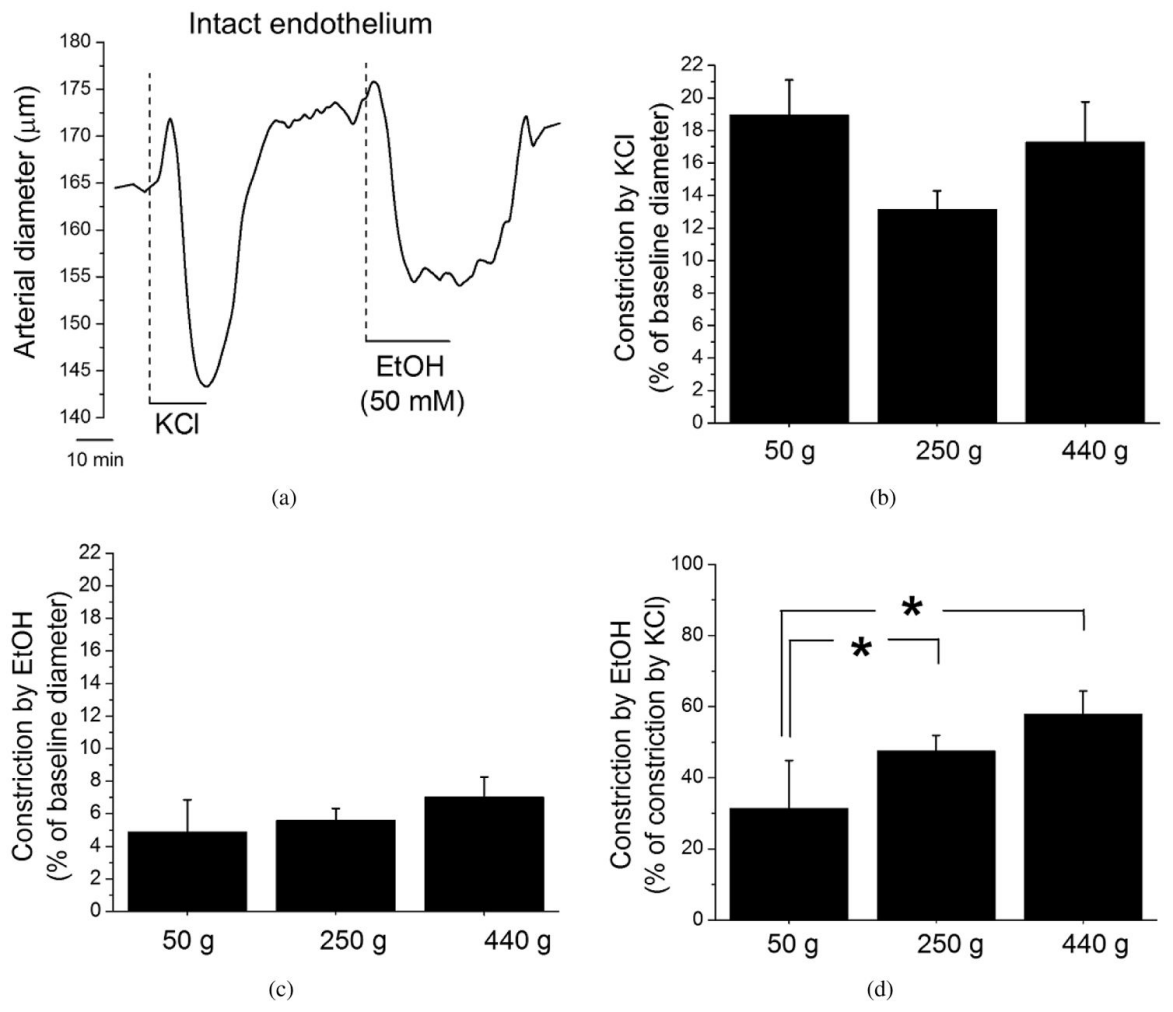
Age-dependent increase in ethanol-induced constriction of rat middle cerebral arteries with intact endothelium. (a) Trace of arterial diameter showing constriction by 60 mM KCl and 50 mM ethanol in pressurized cerebral artery from 250 g rat. Here and in Figures 2 and 7, EtOH: ethanol. Averaged cerebral artery constriction by KCl (b) and ethanol (c) in cerebral arteries with intact endothelium 50 g (*n* = 5), 250 g (*n* = 5), and 440 g (*n* = 8) rats. (d) Averaged cerebral artery constriction by ethanol normalized to constriction by KCl. ^*^P < .05.

Following artery cannulation and myogenic tone development at 60 mm Hg, arteries from the different age groups (see Section 2) were probed with 60 mM KCl to assess maximal arterial constriction due to depolarization (Figure 1(a)). After complete washout of KCl, arteries were tested with 50 mM ethanol. This concentration corresponds to 0.23 mg/dL alcohol in the blood and is reached during moderate-to-heavy episodic drinking in humans [28]. Ethanol was applied for 15–20 min to evoke a steady arterial constriction, yet not long enough to damage arterial tissue. This episodic exposure of cerebral arteries to ethanol also allowed us to compare current results with our previous studies using the same experimental model [13, 14].

KCl-induced cerebral artery constriction remained similar in all three age groups (Figure 1(b)). Ethanol-induced constriction per se showed some tendency to increase with age, yet there were no statistically significant differences when comparing all three experimental groups (Figure 1(c)). However, individual ability of each artery to constrict (as determined by the effect of 60 mM KCl) should be taken into account. Thus, ethanol-induced constriction was normalized to maximally-evoked constriction by KCl. All three age groups differed significantly (Figure 1(d)), with the highest constriction by ethanol being detected in the 440 g group. Thus, susceptibility of cerebral arteries to ethanol-induced constriction increases with age.

### 3.2. The increase in ethanol-induced constriction with age is independent of the presence of a functional endothelium

**Figure 2:**
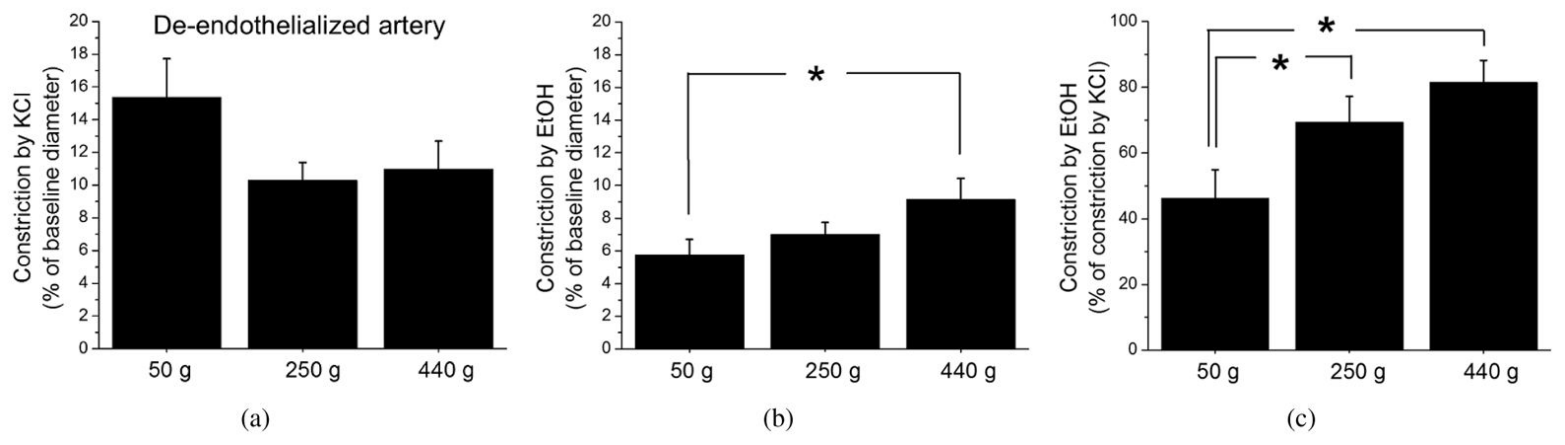
Age-dependent increase in ethanol-induced constriction of rat middle cerebral arteries without endothelium. Averaged cerebral artery constriction by 60 mM KCl (a) and 50 mM ethanol (b) in de-endothelialized cerebral arteries from 50 g (*n* = 10), 250 g (*n* = 21), and 440 g (*n* = 10) rats. (c) Averaged cerebral artery constriction by ethanol normalized to constriction by KCl. ^*^P < .05.

It is known that the endothelium undergoes age-dependent functional changes [29, 30]. Thus, to determine whether the observed age-dependent increase in ethanol-induced cerebral artery constriction is associated with a decline in endothelial function, KCl and ethanol were tested on de-endothelialized arteries from the three age groups. Artery de-endothelization and functional verification of the absence of functional endothelium were confirmed as described in Section 2, and previously standardized by our group [13, 15]. KCl-induced cerebral artery constriction remained similar in all three age groups (Figure 2(a)). As in arteries with intact endothelium, ethanol-induced constriction per se showed a trend to increase with age. Moreover, ethanol-induced constriction in the 440 g group was significantly larger than in the 50 g group (Figure 2(b)). Similar to the results from arteries with intact endothelium, ethanol-induced constriction normalized to maximally-evoked constriction by KCl differed significantly between all three age groups (Figure 2(c)). The highest constriction by ethanol was detected in the 440 g group. Thus, age-dependent increase in artery susceptibility to ethanol-induced constriction was similar between arteries with intact versus denuded endothelium. Therefore, the observed phenomenon of an age-dependent increase in artery susceptibility to ethanol-induced constriction does not involve changes in endothelial function.

### 3.3. Possible factors contributing into the age-dependent susceptibility to ethanol-induced constriction

**Figure 3:**
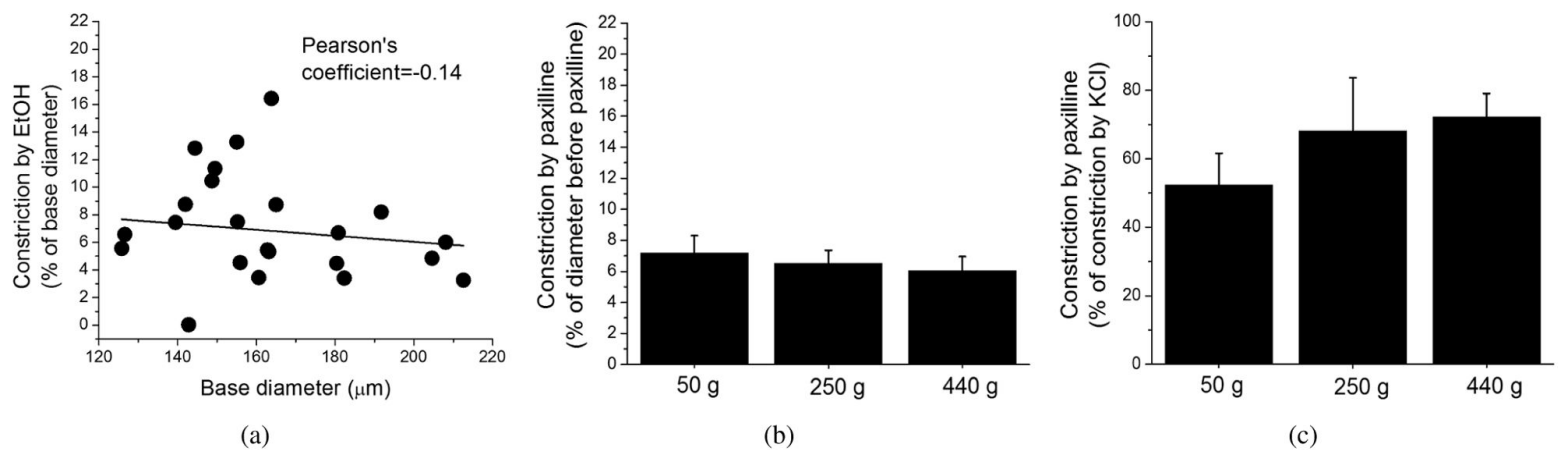
Age-dependent susceptibility of cerebral arteries to ethanol-induced constriction does not correlate with basal cerebral artery diameter or constriction by the BK channel blocker paxilline. (a) Scattered plot showing lack of correlation between constriction of de-endothelialized middle cerebral arteries by ethanol and artery diameter before ethanol application in all three age groups. (b) Averaged paxilline-induced constriction of de-endothelialized middle cerebral arteries from 50 g (*n* = 9), 250 g (*n* = 14), and 440 g (*n* = 6) rats. (c) Averaged constriction of de-endothelialized middle cerebral arteries by paxilline normalized to the corresponding constriction by 60 mM KCl.

As the age of artery donors is increased, so is the average diameter of cerebral vessels. To determine whether larger cerebral arteries constrict more in presence of ethanol, we plotted ethanol-induced constriction against base diameter (e.g., diameter after complete recovery from KCl, immediately before ethanol application) of the de-endothelialized arteries. A scattered graph that includes data from all three age groups is presented in Figure 3(a). Pearsons’s coefficient of the plot is below 0.5; thus, there is no correlation between artery diameter and degree of ethanol-induced constriction.

In vascular smooth muscle, BK channels represent one of the major targets of ethanol. To assess the presence of functional BK channels within rat cerebral arteries from different age groups, we used a selective blocker of BK channels, 1 *μ*M paxilline [14, 31]. We did not detect statistically significant difference in paxilline-induced constriction of de-endothelialized cerebral artery responses between the age groups (Figures 3(b) and 3(c)). These data suggest that functional BK channels were present in arteries of all three age groups.

**Figure 4:**
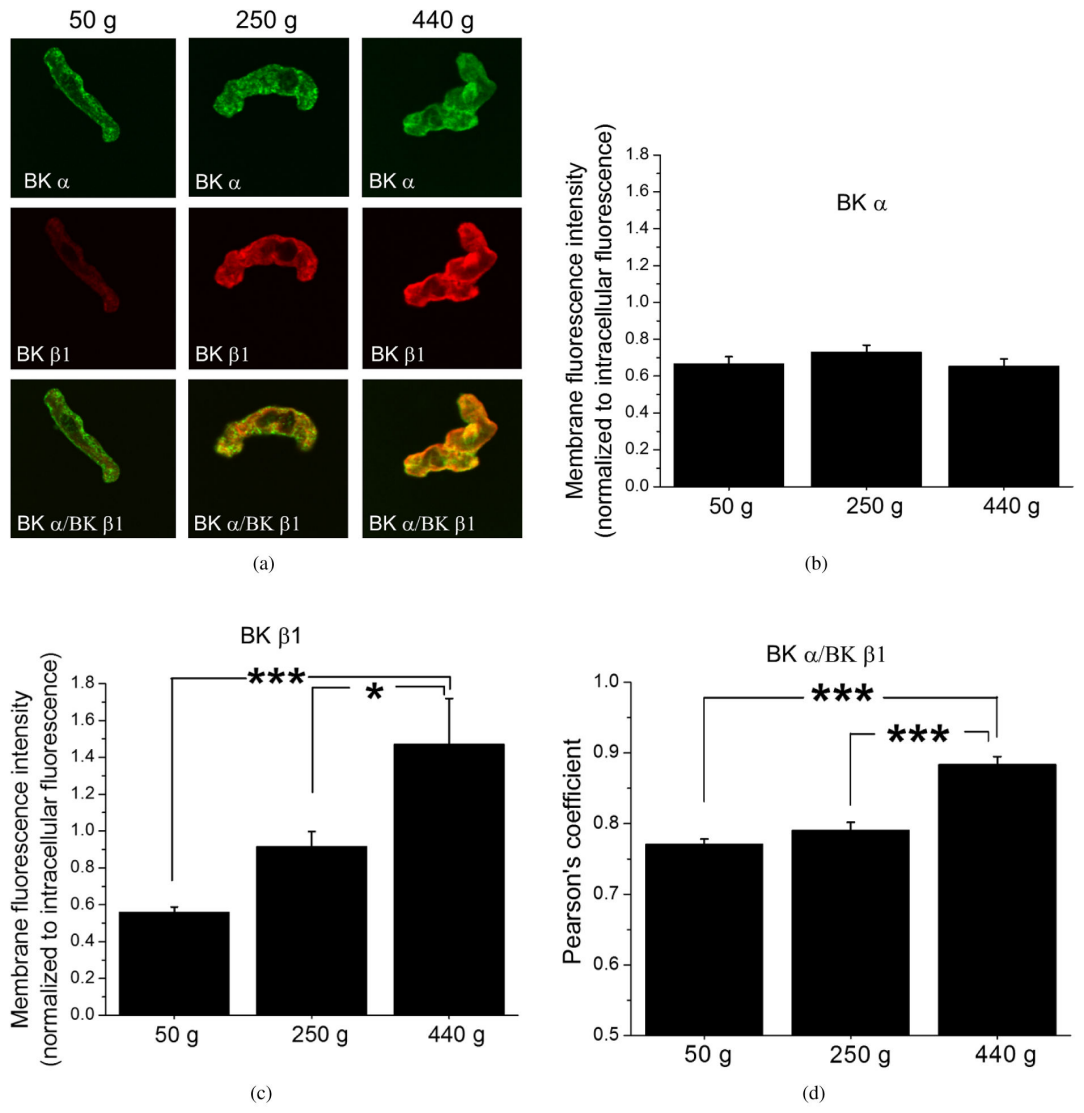
Age-dependent increase in BK *β*1 subunit membrane protein level and colocalization with BK *α* subunit in myocytes freshly isolated from rat middle cerebral arteries. (a) Representative snapshots of confocal imaging of isolated myocytes following immunofluorescence staining against BK *α* and *β*1 subunits. Averaged data showing lack of difference between BK *α* protein level (a), but a progressive increase in BK *β*1 subunit membrane level (b) in middle cerebral artery myocytes from 50 g (*n* = 27), 250 g (*n* = 28), and 440 g (*n* = 25) rats. Data were collected from two rats in each group. ^*^P < .05; ^*^P < .0001. (d) Averaged data showing age-dependent increase in Pearson’s coefficient corresponding to BK*α*-*β*1 subunit colocalization.

The presence of BK *β*1 subunit within the smooth muscle BK channel complex is required for ethanol-induced constriction at physiological conditions: indeed, ethanol-induced inhibition of the channel and eventual constriction are both blunted in cerebral arteries from *KCNMB1* (*β*1-lacking) knock-out mice [14]. Thus, we hypothesize that the progressive increase in ethanol-induced constriction of cerebral arteries with age reflects increasing amounts or functional BK *β*1 protein in the cerebral artery myocyte itself. To test the hypothesis, we performed immunofluorescence staining of freshly isolated cerebral artery myocytes of rat against BK pore-forming *α* and BK accessory *β*1 subunits. Consistent with artery diameter data showing no significant changes in paxilline-induced constriction of the arteries as the rat age increased (Figures 3(b) and 3(c)), immunofluorescence staining against BK *α* subunit failed to detect age-dependent differences in membrane presence of BK *α* when compared to intracellular areas of the myocytes (Figures 4(a) and 4(b)). In sharp contrast, BK *β*1 subunit membrane presence was progressively increased as rats grew older (Figure 4(c)). Moreover, the increase in BK *β*1 subunit protein level was accompanied by an increased colocalization with slo1, which was evident by a significant increase in Pearson’s correlation coefficient between BK *α*- and *β*1-associated fluorescence signals from membrane areas of cerebral artery myocytes of growing rats (Figure 4(d)).

**Figure 5:**
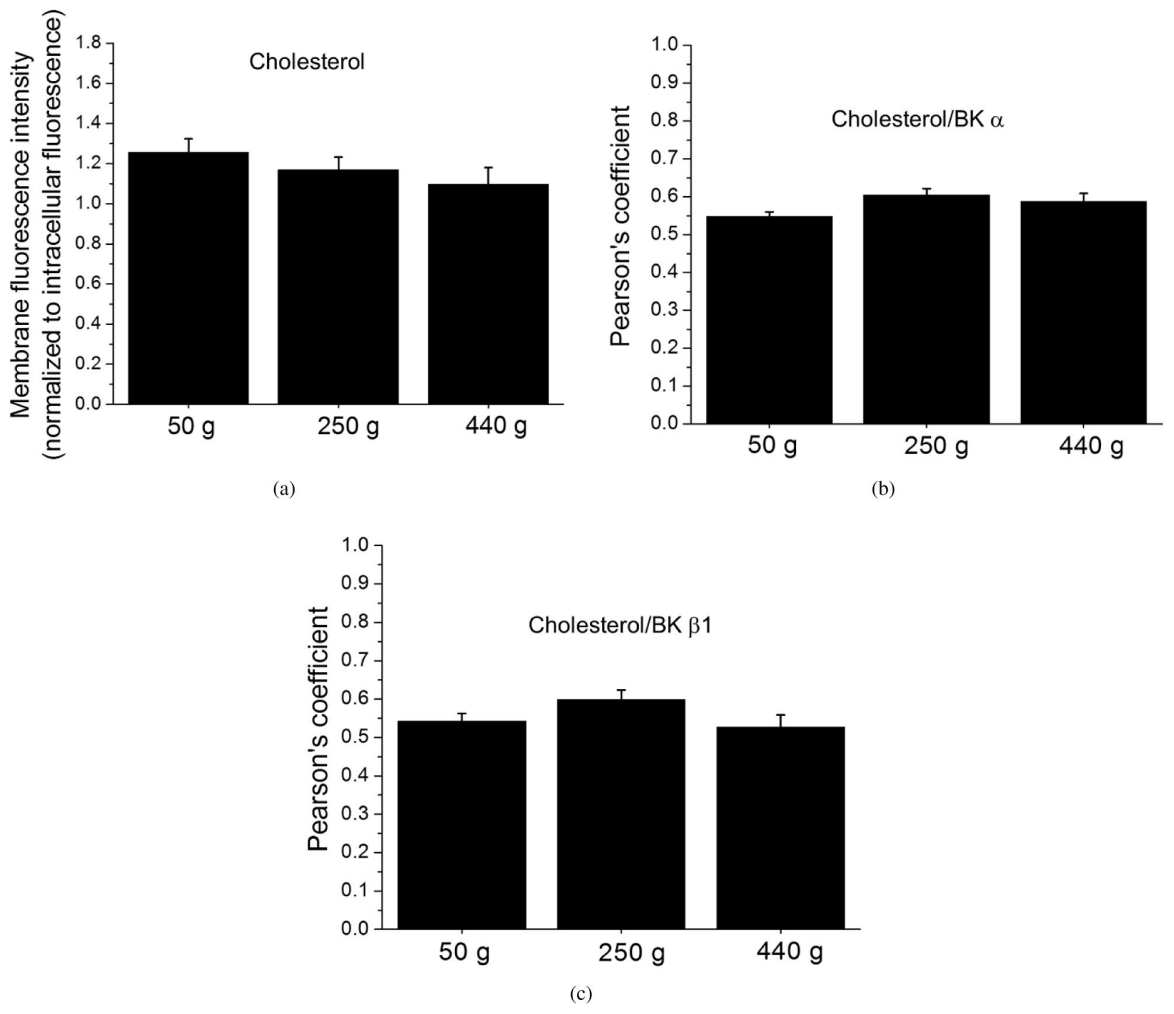
Lack of age-dependent changes in middle cerebral artery myocyte cholesterol level as detected by fluorescence labeling with filipin. (a) Averaged data showing lack of difference between cholesterol-sensitive immunofluorescence (filipin) signal from cerebral artery myocytes of 50 g (*n* = 27), 250 g (*n* = 28), and 440 g (*n* = 25) rats. Data were collected from two rats in each group. Averaged data showing lack of age-dependent changes in Pearson’s coefficient corresponding to BK *α* (b) and *β*1 (c) subunit colocalization with the membrane cholesterol.

The final factor that we considered as a possible contributor to age-dependent increase in artery susceptibility to alcohol-induced constriction was the cholesterol level in the vascular smooth muscle, as we showed that cholesterol levels critically controlled the cerebral artery response to ethanol [15, 32]. Fluorescence staining of freshly isolated cerebral artery myocytes with the cholesterol-sensitive dye filipin failed to detect any significant differences in membrane filipin-associated fluorescence signal in myocytes from all three age groups (Figure 5(a)). Similarly, there were no statistically significant variations in BK *α* or *β*1 subunit colocalization with membrane cholesterol as quantified by a Pearson’s coefficient between BK *α*- and filipin-associated signals (Figure 5(b)), as well as between BK *β*1- and filipin-associated signals (Figure 5(c)).

In summary, out of the several factors that were considered, both an increase in BK *β*1 protein levels and in colocalization of BK *β*1 with BK *α* subunit correlated with the increased susceptibility of cerebral arteries to alcohol-induced constriction (Figures 1(d), 2(c), 4(c), and 4(d)).

### 3.4. Age-dependent increase in BK *β*1 protein level and increased colocalization of BK *β*1 with BK *α* subunit result in age-dependent change of *functional and pharmacological* properties of vascular smooth muscle BK channel

**Figure 6:**
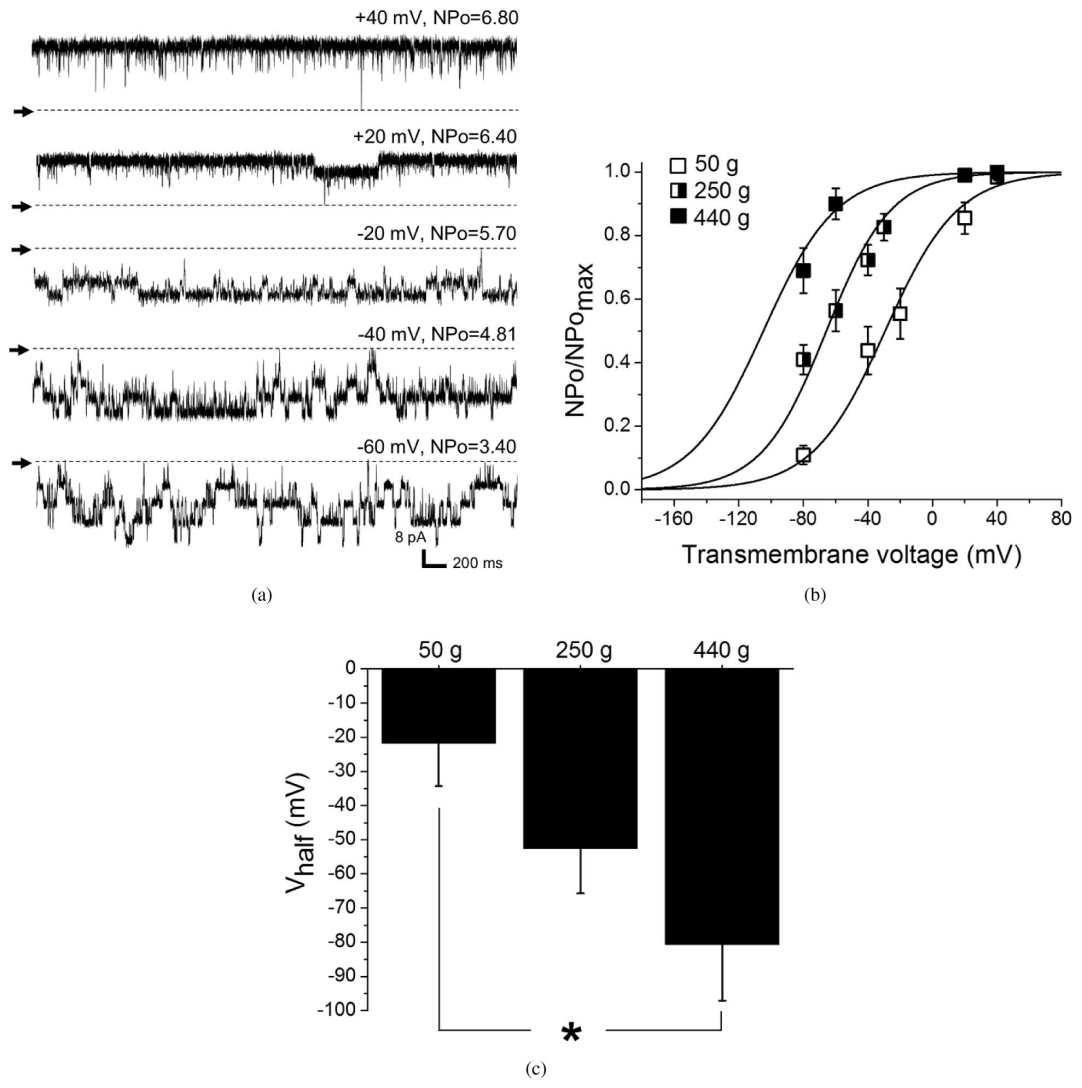
Age-dependent increase in BK channel activity evaluated by patch-clamp recording of native BK channel activity in middle cerebral artery myocytes membrane patches. (a) Examples of patch-clamp recordings of BK channel activity in middle cerebral artery myocyte membrane patch using the inside-out configuration at different holding voltages. Ca^2+^_free_ = 30 *μ*M. (b) Activity-voltage plots showing a progressive shift to the left as age of the rat myocyte donors increases (50 g, *n* = 5; 250 g, *n* = 6; 440 g, *n* = 8). (c) Averaged data showing age-dependent increase in V_half_ (e.g., voltage needed to reach half-maximal activity) of the BK channel.

To ensure that immunofluorescence imaging reflected changes in the *functional* BK *β*1 subunit protein level, we used conventional patch-clamp recordings of native BK channels from freshly isolated cerebral artery myocytes of rats from the different age groups. We used inside-out patches with Ca^2+^_free_ in the bath solution set at 30 *μ*M. This concentration of Ca^2+^ is reached in the vicinity of cerebral artery myocyte BK channel during myocyte constriction and is known to favor ethanol-induced inhibition of the cerebral artery BK channel [14, 33]. BK channel activity was recorded at several voltages, and activity/voltage plots were used to derive V_half_, that is, the transmembrane voltage needed to reach half-maximal open probability of the channel in each membrane patch [34]. Our data show that activity/voltage plots progressively shifted to the left (e.g., towards lower voltages) as rats grew older (Figures 6(a) and 6(b)). This shift towards higher BK channel activity with age was clearly reflected by a statistically significant decrease in V_half_ with age (Figure 6(c)). This increase in basal channel activity with age is consistent with the role of BK *β*1 subunits in enhancing the apparent calcium sensitivity of the channel and favoring higher activity at physiological voltage and Ca^2+^_i_ levels [23].

**Figure 7:**
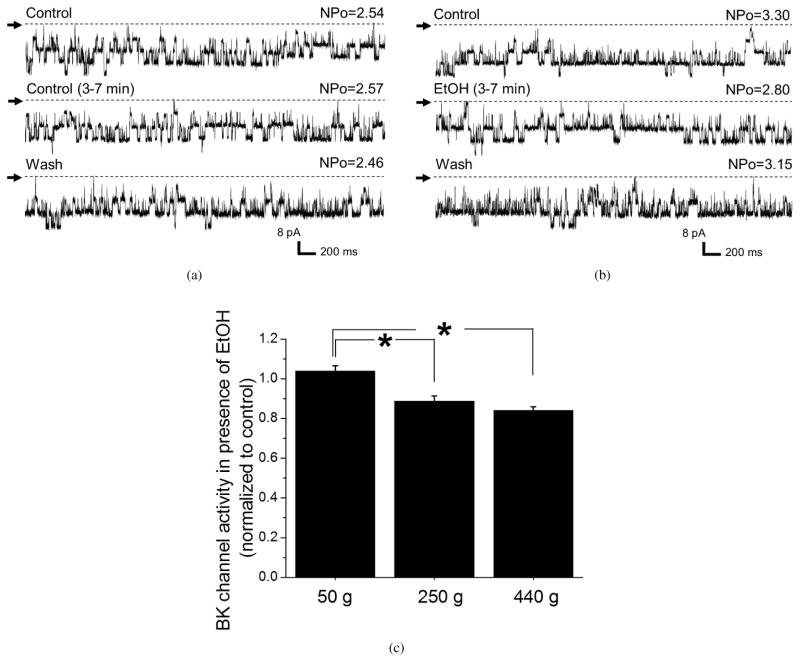
Age-dependent increase in ethanol-induced BK channel inhibition. Original records from the inside-out patches excised from middle cerebral artery myocytes showing lack of change in BK channel activity during perfusion with control bath solution (a), but robust drop in BK channel activity in presence of 50 mM ethanol (b). In (a) and (b), channel activity is shown before and during exposure to either ethanol-free (control bath) or 50 mM ethanol solution. Channel openings are shown as downward reflections; arrow points at the baseline (all channels closed), indicated by a dotted line. Recordings were performed at −40 mV and Ca^2+^_free_ = 30 *μ*M, that is, voltage and calcium levels that mimic physiologic conditions in the vicinity of cerebral artery myocyte BK channel [33, 35]. (c) Averaged data showing a progressive increase in ethanol-induced BK channel inhibition in middle cerebral artery myocytes of 50 g (*n* = 5), 250 g (*n* = 5), and 440 g (*n* = 6) rats. ^*^P < .05.

Remarkably, age-dependent changes were not only limited to basal channel activity; they were also detected in the BK channel sensitivity to ethanol-induced inhibition. As rats grew older, the degree of ethanol-induced BK channel inhibition increased significantly from virtually no effect in myocyte membrane patches of 50 g rats to up to 25–30% inhibition in 250 g and 440 g groups (Figure 7). This increase in ethanol-induced BK channel inhibition with age is consistent with the increased susceptibility of cerebral arteries from ageing animals to ethanol-induced constriction (Figures 1(d) and 2(c)).

## 4. Discussion

In the present study, we demonstrate for the first time an age-dependent increase in the susceptibility of the cerebral arteries to ethanol-induced constriction. This increase is paralleled by the increase in the levels of BK channel *β*1 protein in cerebral artery myocytes and in the degree of ethanol-induced BK channel inhibition in membrane patches from cerebral artery myocytes themselves.

Several clinical and basic research studies have repeatedly shown age-dependent differences in the vulnerability to excessive alcohol consumption and associated end-target organs [36]. For instance, older rats showed increased vulnerability to motor and cognitive impairment by alcohol than that of young adults [37]. In contrast, adolescent brain seemed to be most vulnerable to ethanol-driven neurotoxicity [38]. In our current work, we used rats of different ages: 3 weeks, 8 weeks, and 15 weeks old, which correspond to preadolescence, adolescence, and early adulthood, respectively [37]. We discovered that even within such a short time-span, there was a differential susceptibility of cerebral arteries to the effect of ethanol; the “window of vulnerability” seems to start during adolescence and lasts into early adulthood. Whether susceptibility to ethanol-induced constriction continues to increase into middle age and the elderly or reaches plateau at some point of late adulthood remains to be determined.

It is widely recognized that the differential effects of ethanol across the lifespan are underlied by age-specific neurological mechanisms, such as age-specific expression of protein kinase C gamma (PKC*γ*) in the nervous system [4], N-methyl-D-aspartate receptor (NMDAR) subunit composition [39], and others [37]. Like any other organ, arteries undergo major functional changes across their lifespan. For example, endothelial function progressively declines as ageing progresses [40]. An age-related decline in BK channel *α* and *β*1 subunit functional expression via a transcript downregulatory mechanism has been previously described in coronary myocytes [41]. However, the same group reported that unlike coronary arteries, MaxiK channels from cerebral arteries of old rats (25–30 months) preserved normal surface expression, function, and *α*/*β*1 subunit ratio when compared to young (3–5 months) animals [42]. In the current study, we demonstrate that before the age of 3–5 months, BK *β*1 subunit protein levels are progressively increased while BK *α* protein level remains unchanged. It would be interesting to perform a comprehensive kinetic analysis of BK channel gating at different time points in life to determine whether all aspects of *β*1 subunit-characteristic regulation of BK channel function are proportionally changing over the lifespan. In addition, the possibility that BK channel protein partners and membrane lipid modulators may also contribute to the changes in the BK channel level and function should be explored.

Cholesterol represents one of the critical membrane lipids that control the degree of ethanol-induced BK channel inhibition and resulting vasoconstriction [15, 32]. Remarkably, cholesterol level in the blood seems to undergo dynamic changes throughout lifespan, with both, no apparent change, increase, and decrease in blood cholesterol level being reported [43, 44]. Whether tissue cholesterol levels follow the trend of cholesterol serum concentration remains unknown. We did not detect age-dependent changes in cholesterol content of cerebral artery myocytes (Figure 5), thus, cholesterol was ruled out as a possible determinant of age-dependent increase in artery susceptibility to ethanol-induced constriction.

Even when the intricate mechanisms that underlie age-dependent change in BK channel activity and function remain to be fully explored, the finding of an age-dependent change of BK *β*1 subunit protein level and function in the smooth muscle gains particular importance in light of recent drug development efforts targeted at BK *β*1 subunit-containing channels for the treatment of such prevalent human pathologies as asthma and hypertension [25, 45, 46]. If arise, the new pharmacotherapies may be highly effective during adulthood, but would have limited efficiency in young subjects.

An interesting finding is the lack of difference in paxilline-induced constriction of the arteries across the three age groups under study (Figures 3(b) and 3(c)). Consistent with a decreased BK channel level and function in older myocytes, the ability of the selective BK channel blocker iberiotoxin to constrict coronary rings was found reduced in older coronary artery specimens [41]. In our study, the levels of BK function were higher in adult rats when compared to younger animals. Thus, we expected an increase in paxilline-induced cerebral artery constriction as BK channel function and, presumably, its contribution to the regulation of arterial diameter is increased in the adult group (440 g) (Figure 6). A possible explanation for the similar values of paxilline block across the three age groups under study may reside in the functional channel states targeted by iberiotoxin and paxilline: paxilline is a closed-channel blocker and loses its binding ability to the channel as BK channel open probability (Po) increases [47]. Our data clearly show that BK channel open probability (in our case, NPo) is increased as rats grow from 50 g to 440 g (Figure 6). Thus, the lack of age-dependent increase in paxilline-induced cerebral artery constriction could be explained by a possible progressive reduction in the binding ability of paxilline to a highly active BK channel in presence of increased amounts of activatory BK *β*1 subunits.

Unlike paxilline-induced constriction, that of ethanol was increased as rats grew older from approximately 3 weeks, then to 8 weeks, and finally to 15 weeks old (Figure 1). This increase did not require the presence of a functional endothelium, as it could be observed in de-endothelialized arteries (Figure 2). Considering that after endothelium removal, the majority of cellular content in the artery is represented by vascular smooth muscle cells [16], the molecular players that enable age-dependent increase in ethanol-induced constriction are very likely located in the vascular myocyte. Indeed, age-dependent increase in ethanol-induced vasoconstriction was paralleled by an age-dependent increase in ethanol-induced BK channel inhibition in cerebral artery myocytes (Figure 7). This increase in ethanol-induced BK channel inhibition was observed in excised membrane patches. Thus, the effectors of age-dependent changes in ethanol response are unlikely to include freely diffusible intracellular signaling molecules. However, we cannot rule out the possibility that the increase in BK *β*1 subunit level and function is not the only mechanism responsible for age-dependent changes in cerebral artery susceptibility to ethanol. In particular, another relevant target of ethanol in cardiovascular system—ryanodine receptor (RyR) type 2—has been recently identified [48]. RyR is responsible for handling of calcium release from intracellular stores and ensures BK channel activation in the vascular smooth muscle [49]. Interestingly, dynamic changes in RyR activity have been recently implicated in ageing [50]. Therefore, the question about any possible contribution of RyR into the age-dependent changes in ethanol-induced constriction of cerebral arteries represents a plausible topic for future studies.

In summary, we demonstrated for the first time an increased susceptibility of cerebral arteries to ethanol-induced constriction in a time period that is equivalent to adolescence and early adulthood, when compared to preadolescence. Although age-dependent increase in BK *β*1 subunit protein level and function paralleled the increase in ethanol-induced constriction and ethanol-induced BK channel inhibition, additional molecular players that could contribute to age-dependent susceptibility of cerebral arteries to ethanol cannot be ruled out. Our study raises the hypothesis that one of the mechanisms leading to the well documented age-dependent effects of alcohol on brain function and resulting behavior is an increased BK *β*1-mediated expression and function in the smooth muscle itself, with consequent constriction of cerebral arteries in response to ethanol.
